# An Apple and Acáchul Berry Snack Rich in Bioaccessible Antioxidants and Folic Acid: A Healthy Alternative for Prenatal Diets

**DOI:** 10.3390/foods13050692

**Published:** 2024-02-24

**Authors:** Rocío Corfield, Mariana C. Allievi, Roy Rivero, Tamara A. López, Oscar E. Pérez, Daniela Salvatori, Carolina Schebor

**Affiliations:** 1 Instituto de Tecnología de Alimentos y Procesos Químicos (UBA-CONICET), Departamento de Industrias, Facultad de Ciencias Exactas y Naturales, Universidad de Buenos Aires, Intendente Güiraldes, s/n, Ciudad Universitaria, Buenos Aires 1428, Argentina; rocio.corfield@di.fcen.uba.ar; 2 Instituto de Química Biológica de la Facultad de Ciencias Exactas y Naturales (UBA-CONICET), Departamento de Química Biológica, Facultad de Ciencias Exactas y Naturales, Universidad de Buenos Aires, Intendente Güiraldes, s/n, Ciudad Universitaria, Buenos Aires 1428, Argentina; mallievi@qb.fcen.uba.ar (M.C.A.); oscarperez@qb.fcen.uba.ar (O.E.P.); 3 Instituto de Ciencia y Tecnología de los Alimentos de Entre Ríos (UNER-CONICET), Facultad de Bromatología, Universidad Nacional de Entre Ríos, J. D. Perón 1154, Gualeguaychú 2820, Argentina; roy.rivero@uner.edu.ar (R.R.); tamara.lopez@uner.edu.ar (T.A.L.); 4 Instituto de Investigación y Desarrollo en Ingeniería de Procesos, Biotecnología, y Energías Alternativas (UNCO-CONICET), Universidad Nacional del Comahue, Buenos Aires 1400, Neuquén 8300, Argentina; daniela.salvatori@probien.gob.ar

**Keywords:** fruit leather, folic acid, whey protein isolate, bioaccessibility, bioactive compounds

## Abstract

A fruit leather (apple and acáchul berry) oriented toward women of reproductive age was developed. The snack was supplemented with an ingredient composed of folic acid (FA) and whey proteins (WPI) to ensure the required vitamin intake to prevent fetal neural tube defects. In order to generate a low-calorie snack, alternative sweeteners were used (stevia and maltitol). The fruit leather composition was determined. Also, an in vitro digestion process was carried out to evaluate the bioaccessibility of compounds with antioxidant capacity (AC), total polyphenols (TPCs), total monomeric anthocyanins (ACY), and FA. The quantification of FA was conducted by a microbiological method and by HPLC. The leather contained carbohydrates (70%) and antioxidant compounds, mainly from fruits. Bioaccessibility was high for AC (50%) and TPCs (90%), and low for ACY (17%). Regarding FA, bioaccessibility was higher for WPI-FA (50%) than for FA alone (37%), suggesting that WPI effectively protected the vitamin from processing and digestion. Furthermore, the product was shown to be non-cytotoxic in a Caco-2 cell model. The developed snack is an interesting option due to its low energy intake, no added sugar, and high content of bioactive compounds. Also, the supplementation with WPI-FA improved the conservation and bioaccessibility of FA.

## 1. Introduction

The low intake of folic acid (FA) by women of reproductive age may compromise their pregnancy outcomes, as well as maternal and infant health. This vitamin deficiency is the main cause of neural tube defects like spina bifida and anencephaly [1]. In general, diet alone has shown to be insufficient to provide the recommended daily intake of FA, because a considerable amount of food folate is lost during food processing and cooking [2]. Also, the World Health Organization (WHO) [3] mentions that in communities where food-based policies are not fully applied so far, FA supplementation should be used to improve the folate status of women before conception and during the first trimester of pregnancy. Before and during pregnancy, it is advised to follow a healthy diet in order to improve mother and fetus wellbeing. Also, an intake of 400 µg of vitamin B9 (FA) is suggested to prevent neural tube birth defects.

Additionally, in order to follow a healthy diet to prevent malnutrition, along with a range of noncommunicable conditions and diseases, the World Health Organization [4] released a series of key recommendations. Among them, the WHO advices to consume at least five portions of fruits and vegetables per day, and to reduce the daily intake of free sugars to less than 10% of the total caloric intake. For this reason, the food industry is currently focused on developing nutritious products than are low in simple sugars [5]. In this line, non-caloric sweeteners are an interesting alternative to replace sugar. The steviol glycosides from *Stevia rebaudiana* (Bertoni) have shown excellent results in the development of sweet foods [6,7,8]. However, Samuel et al. [9] recommended that, when replacing sugar with stevia, consideration should be given to the combination with bulking agents such as maltodextrin, polyalcohols, or hydrocolloids to mimic the characteristics of sugar and to provide the moisture and texture that are achieved with regular sugars. In this context, maltitol is an option as it offers various technological advantages [10,11], it is not cariogenic, it is safe for diabetics, and it provides half the calories of sucrose [12].

A way to encourage fruit consumption and lower sugar intake may be the incorporation of fruit-based snacks sweetened with alternative sweeteners. In this sense, fruit leathers are a suitable option to replace natural fruits and increase the intake of vitamins, minerals, fiber, and several phytochemicals [13].

Taking this into account, a fruit leather supplemented with FA was developed, with the aim of offering a practical and appealing sweet snack for women of reproductive age, featuring the advantages of being low in fat and free from added sugars. In a previous work [14], our research group developed a fruit leather composed of apple and the berry acáchul (*Ardisia compressa* Kunth). This leather was rich in bioactive compounds, and it was highly accepted by a sensory test. In addition, our research group developed stable FA ingredients through the formation of complexes between whey proteins and folic acid [15,16]. Considering this background, an apple–acáchul leather, supplemented with our previously designed FA ingredient, was developed.

In order to obtain the health advantages of nutrients and bioactive compounds, they have to become bioaccessible and then bioavailable. Therefore, the evaluation of the bioactive compounds directly in food is not enough to determine their possible effects in vivo, since the compounds have to undergo a complex digestion process [17]. In this sense, there are in vitro methodologies, like the simulation of gastrointestinal digestion, that facilitate the estimation of the bioaccessibility of bioactive compounds and nutrients upon their release from the food matrix [18]. Most in vitro digestion protocols cover the first three stages of digestion—the oral, gastric, and intestinal phases (considering only the small intestine)—but may also include intestinal fermentation studies and the evaluation of intestinal transport using cell models [19,20,21]. There are numerous in vitro digestion assays with varied experimental conditions (enzymes, concentrations, pH, times), which makes it difficult to compare their results. For this reason, based on an international scientific consensus, the first standardized protocol called INFOGEST was developed [21] where existing methodologies were harmonized, and validations were carried out with in vivo digestion models.

In addition, after digestion, bioaccessible compounds with beneficial health effects as well as unwanted compounds can be obtained [22]. In this sense, the possible toxic effect of the digestion products of a designed food can be evaluated using cellular models. Among the cell lines, the human colon adenocarcinoma line (Caco-2) stands out because it has the ability to differentiate into enterocytes and simulate the intestinal epithelium, becoming a widely used tool to study the absorption and transport of analytes of interest, as well as their cytotoxicity [22,23,24,25].

In this context, this study focuses on a comprehensive analysis of the bioaccessibility of potentially bioactive compounds from the developed fruit leather, as well as on evaluating the stability and bioaccessibility of FA within the food system and, finally, evaluating the post-digestion cytotoxicity of this new food in order to show that it consists of a safe food for consumption.

## 2. Materials and Methods

### 2.1. Materials

Acáchul powder was provided by Agroindustrial Food of the Technological University of Xicotepec de Juárez (Puebla, México). The obtaining process consisted of (1) manual harvesting of the ripe acáchul fruits, (2) washing and disinfection with NaClO for 5 min, (3) pulping and drying in a San-Son HCC convection oven (Mexico City, Mexico) at 60 °C for 20 h, and (4) grinding the dehydrated pulp and peel with a Moulinex grinder (Alençon, France) until obtaining a homogeneous powder. Prior to its use, a physicochemical characterization was performed [14]. The powder presented a water activity (a_w_) of 0.18 ± 0.04 and 2.7 ± 0.10% moisture.

Apples (*Granny Smith* var.) from Río Negro Province (Argentinian Patagonia) were purchased from a local market. The apples were washed, peeled, and cut in half, the seeds were removed, and each half was cut into quarters and stored in plastic bags at −20 °C until use. At the time of use, they were thawed and processed with a mixer until obtaining a puree with the following characteristics: soluble solids: 13.5 ± 0.4 °Bx at 25 °C; pH 3.85 ± 0.02 at 25 °C; and total acidity: 0.6 ± 0.003 mg of malic acid/100 g of puree.

For the FA ingredient (WPI-FA), whey protein isolate (WPI) >90% protein was used, which was provided by Arla Foods Ingredients S.A. (Buenos Aires, Argentina), folic acid (FA) (99.0% pure) was kindly donated by Laboratorios Bagó (La Plata, Argentina), and citric acid (food-grade, >90% purity) was used.

The food-grade sweeteners used in the fruit leather formulation were stevia powder (DULRI, Buenos Aires, Argentina) and maltitol 65% (g/100 g) syrup (Ferromet, Buenos Aires, Argentina).

All the reagents used in the determinations were of analytical grade.

### 2.2. Methods

WPI-FA: the ingredient was prepared according to Corfield et al. [15]. Briefly, equal amounts of aqueous solutions of WPI and FA, both at the same concentration (10% *w/w*) and pH 7.0, were mixed. Next, the mixed WPI-FA solution was brought to pH 3.0 with the required volume of concentrated citric acid solution, and subsequently dehydrated using an Alpha 1-4 LD/2-4 LD-2 lyophilizer (Martin Christ, Gefriertrocknungsaniagen GmbH, Osterode, Germany) at a temperature of −54 °C and a pressure of 4.0 Pa for 48 h, obtaining a final % moisture of 2.0 ± 0.3. The controls consisted of solutions of single protein (WPI) and single folic acid (FA) at the respective concentrations, dehydrated by lyophilization under the same conditions as WPI-FA, obtaining final products with a similar % moisture.

Fruit snack: fruit leathers were prepared according to Vázquez-Sanchez et al. [14] and supplemented with the WPI-FA ingredient detailed previously. The procedure consisted of the following steps: (1) Firstly, we mixed the ingredients (Table 1) using a mixer (Phillips, HR1362/02, Eindhoven, The Netherlands).

It should be noted that the amount of the WPI-FA ingredient was calculated in order to provide 400 ppm of FA in each piece of snack (4.4 g of dehydrated product). (2) Secondly, we molded the mixture into 6 cm diameter glass plates; (3) next, it was dehydrated in a convection food dehydrator (FA-10MZ, Maquinarias Cobo, Buenos Aires, Argentina) for 6 h at 60 °C and an air velocity of 1–1.3 m/s. The drying conditions were previously determined [14] in order to meet the desired physicochemical and sensory expectations. The dehydration process was completed when an a_w_ of 0.45 was reached to ensure microbiological safety. (4) Finally, the mixture was demolded and stored in clear zip-lock polypropylene bags at 25.0 ± 0.1 °C, protected from light. The supplemented fruit leather was named L-WPI-FA. Control fruit leathers were also prepared with FA (LC-FA) or WPI (LC-WPI) for FA evaluations.

#### 2.2.1. Composition Characterization of the Supplemented Fruit Leather

The percentage composition of the supplemented fruit leather was carried out following the official methodologies of the AOAC [26]: proteins by the Kjeldahl method (AOAC 984.13), crude fiber by the gravimetric method (AOAC 962.09), fat content by the Soxhlet method (AOAC 960.39), and ash content by the direct method (AOAC 923.03) were analyzed. The carbohydrate content was calculated as the difference between 100 and the sum of the protein, fat, fiber, moisture, and ash contents [27]. The results are expressed in g/100 g of sample as the average ± the standard deviation.

The total energy was calculated as the sum of the energies provided by the different nutrients [27]. In the case of proteins and carbohydrates, the mass was multiplied by a factor of 4, and in the case of fats, the factor 9 was used. The results are expressed in Kcal/100 g of product.

#### 2.2.2. Moisture and Water Activity (a_w_)

The moisture percentage of the supplemented fruit leather was determined by gravimetry using a vacuum oven Fistreem (Cambridge, UK) at 100 °C up to a constant weight. The results were expressed as g of H_2_O/100 g of sample as the mean ± the standard deviation.

The a_w_ was determined by the official method (925.09) [26] using an Aqualab 3TE dew point hygrometer (Decagon Devices, Pullman WA, USA). The results are expressed as the mean ± the standard deviation.

### 2.2.3. In Vitro Gastrointestinal Digestion

The in vitro digestion was carried out in order to find out the bioaccessibility of the potentially bioactive compounds with an antioxidant capacity, like polyphenols and monomeric anthocyanins, as well as the final concentration of FA and its bioaccessibility. In addition, the cytotoxicity of the digested leather could be determined using a human epithelium model made up of Caco-2 cells.

For the in vitro digestion assay, oral, gastric, and small intestinal digestion phases were simulated according to the international standardized protocol INFOGEST [21]. In all phases, samples were incubated at 37 °C in a Function Line 7000 drying stove (Heraeus, Hanau, Germany) and under constant agitation with an orbital shaker, the 304 Vicking M-23 (Vicking SRL, Buenos Aires, Argentina), at 100 rpm. For the oral phase, 5 g of the sample was mixed with 3.5 mL of a simulated salivary fluid (SSF) solution, 0.50 mL of a 1500 U mL^−1^ salivary α-amylase solution, 25 μL of 0.3 M CaCl_2_, and 975 μL of distilled water. The samples were incubated for 2 min. Following this, the gastric phase continued with the addition of 7.5 mL of a simulated gastric fluid (SGF) solution, 1.6 mL of a pepsin solution of 25,000 U mL^−1^, and 5.0 μL 0.3 M CaCl_2_. The pH was adjusted to 3.0, and the volume was increased to a final 10 mL with double-distilled water. The mixture was incubated for 2 h. Afterwards, for the intestinal phase, 11 mL of a simulated intestinal fluid (SIF) solution, 5.0 mL of a pancreatin solution of 800 U mL^−1^, 2.5 mL of a bile salts solution, and 40 μL of 0.3 M CaCl_2_ were added. The pH was adjusted to 7.0, and the volume was increased with double-distilled water until reaching a total of 20 mL. This mixture was incubated for 2 h. After digestion, the supernatant was separated from the insoluble fraction by centrifugation (Eppendorf centrifuge 5408R, 13,000× *g*, 4 °C, 10 min), and the antioxidant capacity as well as the content of total polyphenols, monomeric anthocyanins, and FA were determined. On the other hand, the insoluble fraction was lyophilized and, subsequently, the antioxidant capacity was evaluated by the QUENCHER method, as described below [7].

### 2.2.4. Determination of Antioxidant Capacity and Bioactive Compounds

#### Preparation of Extracts

An amount of 0.6 g of each sample (supplemented fruit leather or soluble fraction from in vitro digestion) was weighed and 3.0 mL of acidified alcohol (85% ethanol with 1.5 N HCl at an 85:15 ratio) were added. The suspensions were shaken for 15 min, protected from light, and then vacuum-filtered with a Büchner funnel. The procedure was repeated three times. Upon completion, the filtered solutions were transferred to 10.0 mL volumetric flasks, and it was brought to volume with acidified alcohol. Three extracts were made for each sample.

#### Total Phenolic Compounds (TPCs)

TPCs were quantified using the Folin–Ciocalteu colorimetric method according to Gagneten et al. [28]. A calibration curve was constructed with gallic acid (R^2^ = 0.999), and TPCs are expressed as mg of gallic acid equivalents (mg of GAE)/g of sample.

##### Antioxidant Capacity (AC)

Radical Discoloration Method (ABTS^•+^)

The methodology consisted of the discoloration of the radical cation (2,2’-azinobis(3-ethylbenzothiazoline-6-sulfonic acid)) (ABTS^•+^). In the case of the ethanolic extracts, we followed Gagneten et al.’s [28] method, and in the case of the lyophilized insoluble fraction obtained from in vitro digestion, the QUENCHER method [7] was applied. In both cases, a calibration curve was constructed with gallic acid (R^2^ = 0.997), and the results are expressed as mg of GAE/g of sample.

Antioxidant Iron Reducing Power (FRAP)

This quantification was performed following the FRAP method [29]. A calibration curve was made with gallic acid (R^2^ = 0.995) and the results are expressed as mg of GAE/g of sample.

##### Total Monomeric Anthocyanins (ACY)

The ACY content was analyzed by the differential pH method [28]. The results are expressed as mg of cyanidin-3-O-glucoside (Cyn-3-glu) equivalents/g of sample.

#### 2.2.5. Folic Acid (FA)

Folic acid was determined in the supernatants obtained after the in vitro digestion (see Section 2.2.3) of L-WPI-FA, LC-FA, and LC-WPI samples, and also in a sample of digested water in order to control model gastrointestinal solutions. Quantification was performed by two methods: the AOAC microbiological method, and by an alternative unofficial method using HPLC [30].

In the official method, the protocol described by Corfield et al. [15,16] was followed. The *Lacticaseibacillus casei* BL23 (*L. casei* BL23) strain was used, which was previously treated in order to exhaust the intracellular FA pool, and FACM (Folic Acid Casei Medium; Difco, Buenos Aires, Argentina) was used as the culture medium. Parallel to the measurement, an FA calibration curve was performed. The determination consisted of inoculating the bacterial suspension of *L. casei* BL23 in different tubes containing the FACM medium and different concentrations of FA. These cultures were incubated for 48 h, and subsequently, the optical density (OD) was measured at 600 nm. Simultaneously, the samples were measured following the same procedure but substituting the FA standard solutions for the samples. The results are expressed in μg of FA/g of sample.

The alternative method was performed following the Koning et al. [30] protocol with some modifications. For these determinations, an HPLC device (Waters 1525, Milford MA, USA) equipped with an automatic injector (Waters 2707, Milford MA, USA) and with a matrix detector photodiode (Waters 2996, Milford MA, USA) was used. A Lichrospher^®^ 100 RP-18e column (250 × 4 mm, 5 µm) was used. The mobile phase was constituted by two portions in pump A (methanol/acetonitrile, 1:1) and in pump B (water/phosphoric acid, 99:1). The elution gradient was optimized as follows: T, time (min)/mobile phase—A:B (%): T0/10:90, T3/10:90, T12.5/15:85, T14/15:85, T15/10:90, and T25/10:90 at a flow rate of 0.8 mL/min and at a stabilization temperature of 30 °C. Both the standard and the samples were filtered with syringe filters of 0.22-micron pore thickness. The volume injected per sample was 40 µL (*n* = 3).

The standard consisted of a 100 ppm FA solution prepared in NaOH 0.05 M. The calibration curve consisted of 6 points from 1 to 50 ppm dissolved in HCl 0.05 M (R^2^ = 0.999). Both the curve and the samples were filtered through syringe filters with a pore thickness of 0.22 µm. The volume injected per sample was 40 µL (*n* = 3). Measurements were made at 290 nm. The bioaccessibility was calculated according to Corfield et al. [16].

#### 2.2.6. Biological Studies: Cytotoxicity

The cellular metabolic activity of Caco-2 cells when exposed to the supernatant of the digested apple–acáchul leathers was evaluated. Caco-2 cells were used for this study because they are widely used as a model in different cytotoxicity studies [31,32]. The MTT test was carried out following the procedure of Domínguez Rubio et al. [24] with some modifications. Briefly, 2 × 10^4^ Caco-2 human adenocarcinoma cells (ATCCs) were subcultured in a 96-well plate using Dulbecco’s Modified Eagle Medium (DMEM) supplemented with 10% heat-inactivated fetal bovine serum (FBS), 50 U/mL of penicillin, and 50 μg/mL of streptomycin. Incubation was carried out at 37 °C with a humidified 5% CO_2_ atmosphere for 48 h to achieve cell adherence and greater than 80% confluency. After this time, the culture medium was changed for the different treatments, which consisted of the post-digestion in vitro L-WPI-FA and water (digestion control) at different concentrations diluted with DMEM 10% FBS: 10:90 (10% digested sample); 20:80 (20% digested sample); 30:70 (30% digested sample); 50:50 (50% digested sample); and 100:0 (100% digested sample). On the other hand, cells treated only with DMEM 10% FBS were considered as positive control. After 24 h of exposure to the treatments, a solution of tetrazolium salt (MTT) was added at a final concentration of 0.5 mg/mL and incubated for two additional hours. Subsequently, the formed insoluble compound (formazan) was solubilized with DMSO and, subsequently, the absorbance was measured at 570 nm and 690 nm (background) using a POLARstar Omega microplate reader (Ortenberg, Germany). The difference between absorption at 570 nm and 690 nm was reported as an indicator of viability. Cells containing only DMEM 10% FBS were considered 100% viable.

#### 2.2.7. Statistical Analysis

Data are shown as the mean ± standard deviation. All assays were performed in triplicate. The results obtained were compared by means of an ANOVA analysis or *t*-test (*p* ≤ 0.05) using the GraphPad Prism 6.0 software (San Diego, CA, USA, 2014).

## 3. Results and Discussion

### 3.1. Composition Characterization

Apple–acáchul leathers supplemented with FA (L-WPI-FA) were characterized. In order to obtain a snack that could be stored at room temperature, the supplemented fruit leathers were dehydrated up to an a_w_ = 0.45 ± 0.1, reaching 18 ± 1% moisture. Other authors reached similar values of a_w_ and % moisture, such as Torres et al. [33], who reported, for apple leathers and apple leathers supplemented with maqui extract, a_w_ values between 0.56 and 0.69, and moisture values between 15.9 and 16.6%. Table 2 shows the centesimal composition of the apple–acáchul leathers supplemented with FA (L-WPI-FA).

Among the analyzed macronutrients, carbohydrates stood out for presenting a higher proportion, while fats and proteins showed the lowest values. These results are related to the fruit materials used in the formulation, since they represent more than 90% of the total ingredients. Contrarily, KC et al. [34] developed different formulations of lapsi fruit leathers, obtaining between 85 and 89% carbohydrates in the composition; however, the formulations contained between 20 and 50% commercial sugar (sucrose), resulting in a product high in carbohydrates but largely due to added sugars. On the other hand, the fruit components contributed an interesting fiber content (Table 2), obtaining values higher than those reported by Ayelew and Emire [35] for leathers composed of *Anona muricata* L. fruit and *Avena sativa* flour; by Ayu et al. [36] for pineapple and okra leathers; and by KC et al. [34] for lapsi fruit leathers. In this regard, it has been shown that a high fiber intake is associated with body weight control, reduced risk of coronary heart disease [37,38,39], the prevention of constipation, control of serum cholesterol absorption, the prevention of colon and intestinal cancer, and the promotion of beneficial microorganisms [40,41].

Additionally, the total energy intake resulted in 284.7 Kcal/100 g of product and 12.2 Kcal per unit of supplemented fruit leather (approximately 4.4 g). Comparable commercial products like “Fruit Snack Frutina” (UK) provides a similar energy intake of 284.5 Kcal/100 g. In a 2000 Kcal/day diet, the consumption of one serving (two pieces of supplemented fruit leather) would provide 1.25% of the recommended daily energy value (%DV). This contribution is mainly due to fruits, since, in these snacks, the fruits provide 82% of the total calories, and the maltitol syrup provides the remaining 18%. Another aspect to highlight is that, considering a 60 kg person, this portion represents only 3% of the acceptable daily intake (ADI) of stevia stipulated by the Joint FAO/WHO Expert Committee on Food Additives [42]. Therefore, this product could be considered an appropriate snack for people who follow low-calorie diets, since it has low caloric value, an interesting contribution of fiber, and nutrients from fruits, and contains a very low percentage of stevia with respect to the ADI value.

### 3.2. Bioaccessibility of Bioactive Compounds and Antioxidant Capacity

**Figure 1:**
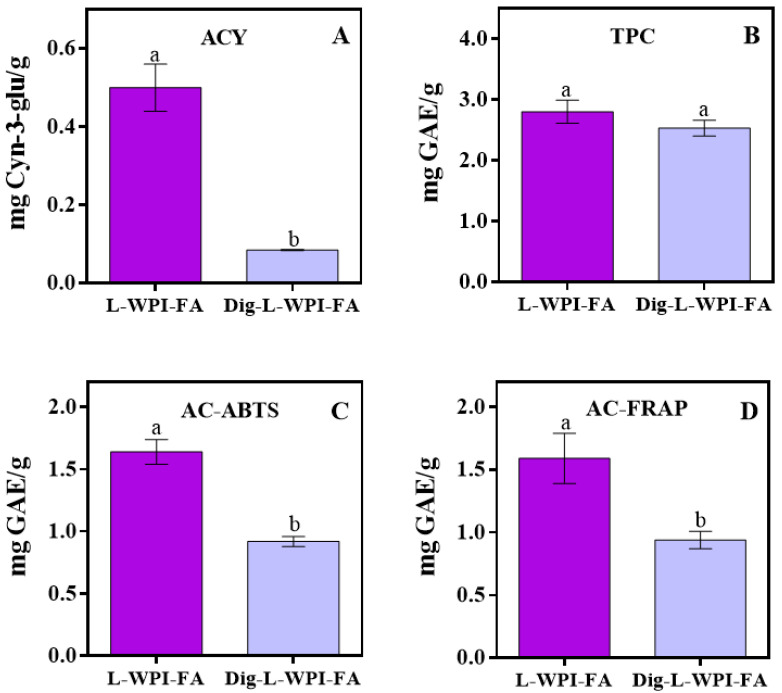
Content of total monomeric anthocyanins (ACY) (**A**), total polyphenols (TPCs) (**B**), and antioxidant capacity (AC) measured by the ABTS (**C**) and FRAP (**D**) methods obtained for undigested (L-WPI-FA) and digested leathers (Dig-L-WPI-FA). Different letters indicate significant differences *p* ≤ 0.05.

The bioactive compounds (ACY and TPCs) and the antioxidant capacity (AC) were determined in the supplemented fruit leather, and also, the bioaccessibility was estimated after a simulation of the gastrointestinal process [21]. Figure 1 compares the results obtained for the undigested leather (L-WPI-FA) and for the leather subjected to in vitro digestion (Dig-L-WPI-FA). In the case of ACY (Figure 1A), a strong decrease occurred upon digestion, reaching only 17% bioaccessibility of these compounds, while TPCs showed 90% bioaccessibility (Figure 1B). This apparent discrepancy could be attributed to changes in anthocyanin molecules during digestion. Similar results were obtained by Gómez-Mattson et al. [43] when studying an elderberry ingredient. According to these authors, the decrease in ACY could be related to the rupture of the anthocyanin ring as a result of the alkaline conditions in the intestinal phase. In this sense, Castañeda-Ovando et al. [44] pointed out that, by increasing the pH of anthocyanins above five, the structure is broken, and when the pH rises above six, chalcones are formed. This would partly explain why the TPC content has been maintained after digestion, since chalcones are flavonoids. However, it should be considered that there may be a balance between degraded polyphenols, those generated by structural changes to the molecules, such as anthocyanins, and those released from the food matrix because of digestion. Regarding AC, a 50% bioaccessibility was recorded, independently of the used method (Figure 1C,D).

Concerning the AC obtained from the insoluble fraction after in vitro digestion, a value of 134.53 ± 10.4 mg of GAE/g of supplemented fruit leather was obtained. This value was much higher than the value obtained in the supernatant of the digested leather (Figure 1C); however, a direct comparison should not be made because the methods to determine AC are different. It is relevant to note that 80% of the digested sample corresponded to the insoluble fraction. These results are in agreement with those reported by Archaina et al. [7], who performed an in vitro digestion of freeze-dried blackcurrant snacks. The presence of this insoluble fraction could be beneficial for the gut microbiota, since recent scientific evidence suggests that certain polyphenols could exert their beneficial action through the modulation of the microbiota, promoting the proliferation of beneficial bacteria [45,46]. Also, according to Wu et al. [47], the antioxidant compounds that reach the colon can be biotransformed and absorbed. The colonic microbiota is responsible for processing most of the unabsorbed flavonoids in the small intestine and transforming them into bioaccessible species [17].

### 3.3. FA Quantification

**Figure 2:**
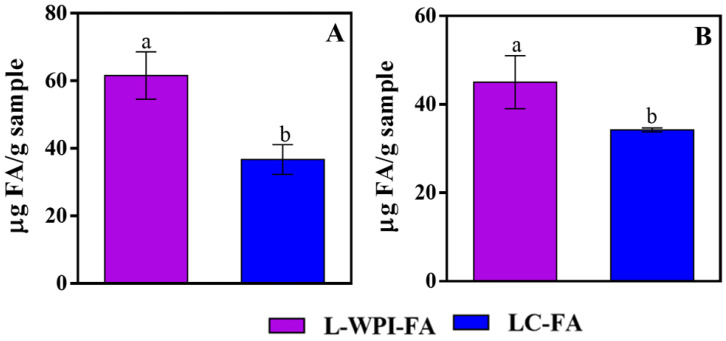
Analysis of the FA present in the supplemented fruit leathers, L-WPI-FA and LC-FA, after in vitro digestion. FA concentration in terms of bioavailability against *L. casei* BL23 (**A**); FA concentration measured by HPLC (**B**). Different letters indicate significant differences *p* ≤ 0.05.

Figure 2A shows the FA contents obtained by the microbiological method. In the case of LC-WPI, it did not show the presence of FA, suggesting that the fruit ingredients of the leather did not have natural folates. When analyzing the leathers supplemented with the WPI-FA ingredient, L-WPI-FA presented a higher FA concentration than LC-FA. In this sense, Corfield et al. [16] compared the FA content of WPI-FA complexes and free FA, both at a concentration of 0.01825% *w/w*, using the microbiological method. These authors observed a higher FA concentration in the complexes, and suggested that the FA mobilized by WPI-FA complexes could be more stable than the free vitamin at different digestion steps. On the other hand, Ochnio et al. [48] developed FA nanocomplexes from soy protein isolates and found that FA quantification by *L. casei* BL23 was higher when the vitamin interacted with globulins. They postulated that FA could be more bioavailable to *L. casei* BL23 when interacting with other molecules. These results are explained by considering that *L. casei* BL23 has its own folate transport system [49,50], but also presents a proteolytic system for the transport of protein products through the cell membrane [51,52], which could be activated for the entry of FA when it was transported in FA–protein complexes. Therefore, the results obtained from the in vitro digestion of the leather supplemented with FA could imply that the vitamin in L-WPI-FA is more stable for the effects of the processing during leather production and in vitro digestion, and/or that in the WPI-FA complex, the vitamin is more bioavailable for this microorganism. This result is interesting considering the improvement of the bioavailability of the vitamin; however, it is still an assumption.

In order to clarify the previous hypothesis, the FA present in the digested leathers was also evaluated by an alternative non-biological method (Figure 2B).

This method also showed that the values obtained for the L-WPI-FA digested system were higher than those obtained for the LC-FA control. This result confirms that FA was more stable when it interacted with WPI, since this method yields an absolute result regarding the quantification of the vitamin. Several authors [53,54] reported a greater resistance of encapsulated FA against different stresses (heat, UV radiation, digestive processes). All of this evidence suggests that the encapsulation of this vitamin is an important solution to protect it from degradation. In this case, the WPI contributed to reduce the degradation of FA during the processing of the supplemented fruit leather (dehydration for 6 h at 60 °C) and, subsequently, to the different steps of the in vitro digestion.

When comparing the results obtained by the official microbiological method (Figure 2A) and the alternative HPLC method (Figure 2B), the FA content in LC-FA did not show substantial differences. However, L-WPI-FA did show differences, obtaining 30% more FA by the microbiological method. In this sense, Koning et al. [55] found differences of up to 25% between the quantification of folates using the microbiological method and the alternative HPLC method, indicating that the causes of this phenomenon were not clear. Also, Kariluto et al. [56] found differences between the quantification of folates in wheat and rye breads by the microbiological method and HPLC, explaining that these differences could be due to the effects of the bacteria. Other works also found differences, but it was highlighted that the foods analyzed contained different forms of folate, which were valued using standards such as 10-HCO folate, 5-HCO-H4 folate, and 5-CH3-H4-folate, among others [30,57]. However, this fact would not explain the results obtained in this work, since the leather without the FA supplement (LC-WPI) did not contain natural folates as determined by the microbiological method and, on the other hand, the vitamin registered in the LC-FA leather was the same for both methods. Therefore, these results could corroborate the hypothesis: the interaction of WPI with FA lead to an increase in the bioavailability of the vitamin for *L. casei* BL23.

#### Bioaccessibility of FA

To evaluate the bioaccessibility of the FA present in L-WPI-FA and LC-FA, the results obtained by the alternative HPLC method (Figure 2B) were considered, since this method is more appropriate to quantify the absolute FA that reaches the intestine at the end of digestion, without showing differences in the quantification related to the bioavailability of the vitamin in the bacteria. It was observed that the vitamin presented a bioaccessibility of 50% in the L-WPI-FA sample and 37% in the LC-FA leather. These results are consistent with those previously discussed, demonstrating once again the contribution in terms of protection and stability that protein provides to the vitamin. However, although the WPI generated protection for FA, it was partial, since the remaining 50% of FA was probably deteriorated. This was expected due to the processing conditions of the supplemented fruit leather and the in vitro digestion steps; therefore, it is reasonable that some degradation of FA had taken place. In this sense, Delchier et al. [58] studied the degradation of folates present in spinach and beans as a function of time and temperature and showed that folate losses were 70% for spinach and 80% for beans when they were exposed to 65 °C for 60 and 90 min, respectively. On the other hand, Liu et al. [59], when studying the bioaccessibility of folate in various flours, found that folate bioaccessibility depended on food matrices, ranging from 42% to 67% in flours.

### 3.4. Cytotoxicity

**Figure 3:**
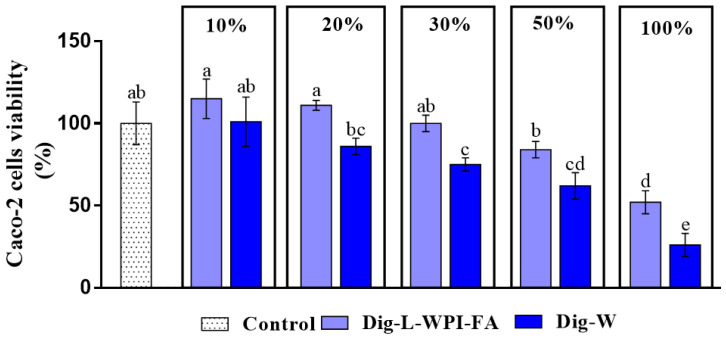
Cell viability of the Caco-2 line exposed to different concentrations of digested (Dig) L-WPI-FA or water (W). The control bar corresponds to 100% cell viability (cells treated only with DMEM 10% FBS). Different lowercase letters indicate significant differences between treatments (*p* ≤ 0.05).

The possible cytotoxic effect of the leathers was assessed by studying the cellular metabolic activity of Caco-2 cells exposed to different concentrations of the digested leathers (Dig-L-WPI-FA) compared to digested water (Dig-W) (Figure 3).

When performing in vitro gastrointestinal digestion following the INFOGEST [21] protocol, bile salts are used, and these components have been reported to be toxic to cells [22]. This effect only occurs in in vitro study models given that, during in vivo digestion, there are specific mechanisms to reabsorb bile salts [60]. Toxic effects were detected for both samples; Dig-W presented cytotoxicity from a 20% concentration, while Dig-L-WPI-FA did not present cytotoxic effects up to 50%. The results obtained for Dig-W show that, in effect, the components of the solutions employed for the in vitro digestion generated a cytotoxic effect on the Caco-2 line. Meanwhile, a protective effect of the supplemented fruit leather components was evident, as a higher viability was observed for Dig-L-WPI-FA compared with Dig-W at all the studied concentrations. In this sense, Miao et al. [61] and Bellion et al. [62] reported a protective effect on Caco-2 cells in studies with apples; a protective effect of different berries has also been reported [63,64]. Therefore, in the case of leathers, both apples and acáchul could generate this positive effect.

## 4. Conclusions

Folic acid-supplemented leathers were developed using an ingredient composed of dairy proteins and folic acid. The centesimal composition analysis revealed that the majority of calories in this snack are attributed to the fruit components, constituting a favorable characteristic for people who follow a healthy diet. In addition, the supplemented fruit leather had an interesting fiber content that could contribute to multiple health benefits. On the other hand, a high bioaccessibility of the antioxidant capacity was found, added to the fact that the insoluble fraction of the in vitro digestion also retained a high antioxidant capacity, suggesting a possible contribution to produce a positive biological effect on the intestinal microbiota.

Regarding FA, the bioavailability study against *L. casei* BL23 after in vitro digestion provided information about the stability of the vitamin throughout the leather processing and during in vitro digestion. On the other hand, the L-WPI-FA leathers obtained greater FA bioavailability than the control ones (L-FA), suggesting that the WPI could protect the vitamin, and at the same time, increase the FA bioavailability evaluated in the lactobacilli model. This hypothesis was corroborated by the quantification of FA by HPLC. In this way, it was possible to determine the bioaccessibility of the vitamin, demonstrating that it has greater bioaccessibility when it is complexed with the protein than when it is in free form. The results obtained from the quantification of the vitamin using the official microbiological method and the alternative HPLC method are a very important precedent for future studies of foods supplemented with FA using ingredients formulated based on proteins.

Finally, cytotoxicity studies made it possible to guarantee the food safety of the digested leather against the Caco-2 model, and additionally showed a protective effect on these cells against the injury produced by bile salts.

The supplementation of this snack with FA makes this product a more attractive alternative for people with high requirements for this vitamin, such as pregnant women, given that, currently, the available options for FA intake are limited to some enriched food products following local guides, or supplements. Additionally, the supplemented fruit leather is devoid of added sugars, gluten, or wheat derivatives, and has a low caloric content. Therefore, from a health perspective, this snack is suitable for individuals with various dietary needs, including diabetics, those with celiac disease or gluten allergies, and individuals adhering to a low-calorie diet. Moreover, in accordance with WHO recommendations, this snack serves as an appealing means of increasing fruit intake.

Future studies will focus on antioxidant compounds and FA absorption studies using the Caco-2 model, as well as FA bioavailability studies on murine models.

## Figures and Tables

**Table 1:** Formulation of fruit leathers supplemented with WPI-FA.

Components	(g/100 g)
Apple puree	90.16
Acáchul powder	1.0
Maltitol syrup	8.5
Stevia	0.33
WPI-FA	0.0074

**Table 2:** Composition of the supplemented fruit leather (g/100 g product). Data represent the mean ± standard deviation.

Composition	Values (g/100 g)
Carbohydrates	70.03 ± 4.6
Crude Fiber	5.29 ± 0.69
Protein	0.88 ± 0.20
Fat	0.12 ± 0.03
Ash	4.70 ± 0.67

## Data Availability

The original contributions presented in the study are included in the article, further inquiries can be directed to the corresponding author.
